# Food and Agriculture Policy in Europe

**DOI:** 10.3934/publichealth.2016.1.131

**Published:** 2016-03-21

**Authors:** Christopher A Birt

**Affiliations:** Department of Public Health and Policy, University of Liverpool

**Keywords:** diet, public health nutrition, risk factors, European Commission, Common Agricultural Policy, public health advocacy

## Abstract

Diet includes many risk factors for the most common non-communicable diseases (NCDs), but diets consumed in Europe and in other parts of the developed world are not being modified sufficiently to take account of health priorities concerning, in particular, the prevention of NCDs, while much excess mortality and morbidity could be prevented by government actions to regulate appropriately both the agricultural and food industries, and to apply appropriate taxes and subsidies to promote healthier nutrition. In Europe, the Common Agricultural Policy (CAP) continues to promote production of saturated fat rich foods and sugar, with scarce attempts to promote increased production of fruit and vegetables. Meanwhile, the food industry continues to market secondary food products rich in sugar, salt and saturated fats. Powerful lobbies seek to block reform; however, necessary reforms are indicated in the interests of improved nutritional health.

## 1. Background

As in other parts of the world, the diets eaten by the peoples of Europe have been determined mostly by history, culture and tradition. However, food policy as we now understand it originates from the late 1940s, in the aftermath of the Second World War. Although often not nowadays remembered, because that War itself destroyed all infrastructure, including farming, towards the end of the War, and for a few years subsequently, there was severe famine and starvation in much of Europe; certainly millions died at that time, although the overall number of deaths has not been properly assessed, and indeed to do so would be very difficult, as so little about this was recorded at the time in any systematic manner; such evidence as we have is anecdotal [1]–[4].

Accordingly, when a degree of organisation was re-established in Europe's main cities, city leaders (e.g. mayors, etc.) called for renewed production of traditional foods, for which the people themselves were crying out: in particular, for milk, bread, meat (especially beef), and sugar. It is therefore not surprising that when farming began to be organised at European level, these were the products that farmers were encouraged to produce. There were earlier fore-runners, but the European CAP, as we now know it, was launched in 1962 [5], [6]; it was one of the very first initiatives of the recently-established European Economic Community (EEC), a for-runner of the European Union (EU).

## 2. Development of the Common Agricultural Policy

In the circumstances, as described above, it is unsurprising that the priorities for the designers of the original CAP were two-fold: firstly, to ensure future food security in Europe, so that starvation such as that experienced in the 1940s should never again be experienced, and secondly, to ensure that the farming community received adequate financial rewards for their work, so as to protect the viability of Europe's rural economies, and to discourage agricultural labourers from leaving the land in the hope of finding higher paid employment in cities. At that time, healthy nutrition, and how farming policy might influence this, was not well understood [7], so it scarcely influenced food and agriculture policy, while food security and an adequate supply of essential nutrients were identified as the necessary health priorities for agriculture, although, as set up in 1962, the CAP was supposed also to promote human health generally[8].

These two priorities were guaranteed and achieved by two aspects of the CAP [5], [6]:

1. by providing farmers with generous production subsidies; these guaranteed larger subsidy payments to match increased production.
2. by guaranteeing minimum prices for targeted products; if and when market prices were to fall below these price levels, the European Commission (EC) was required to buy such unsold products at these guaranteed price levels.

It is not surprising that Europe's farmers responded by increasing farm production rapidly, year by year; thus by the 1970s food surpluses began to accumulate, when production exceeded consumption. Europe's press began to refer to “beef mountains” (in reality held in cold storage facilities), “milk lakes”, etc., and various measures were introduced as attempts to limit these food surpluses, such a milk quotas for farmers, whereby farmers were penalised if they over-produced milk beyond what their quotas allowed [9],[10].

Sugar posed a particular problem. If free trade across the world were to operate, no beet sugar production would take place in Europe, as sufficient quantities of cane sugar could be provided more cheaply from tropical and sub-tropical countries. However, beet sugar production had been encouraged and supported in both World Wars (to ensure within-country self-sufficiency), so after the Second War, sugar farmers expected such support to continue. So to provide this within the CAP an artificial sugar economy had to be created, with the European sugar price being kept higher than the world price, so that farmers could still make profits from farming sugar beet (they were also receiving their production subsidies). As in the case of other products, over-production resulted, so that the EC was forced to sell Europe's excess sugar production on the world market; the EC had to buy this sugar at the European minimum price level, and sell it at the much lower world price level (therefore at a loss). However, excess production in Europe was so great that the world market prices were depressed by the flood of European beet sugar, which caused economic problems for producers in some cane sugar producing countries [11]. This problem has at last been addressed in the recent CAP reforms introduced in 2014; the distortions to the market have already been much reduced and should have been eradicated by the end of 2017; it is anticipated that sugar beet farmers will find new markets for beet (e.g. it would be sold for conversion to biofuels) [12].

Following several previous attempts to reduce overproduction of subsidised farm products (e.g. by limiting milk production by providing farmers with milk quotas, which could not be exceeded [13]), production subsidies were ended from 2005, since when farmers have received “whole farm subsidies” similar in size to what they received previously, but without any link to production: farmers receive subsidies if they use the land for various approved types of farm production [14], and provided they maintain basic minimum standards of rural environmental protection, of animal husbandry, and of food safety; however, within these reforms there were no incentives to alter the products to be farmed (except that from 2008 fruit and vegetables were added to the list of permitted farm products within whole farm subsidy arrangements); public health nutrition was still not a stated priority [15],[16]. It is worthy of note that European agriculture has a very powerful and effective lobby in Brussels, and many MEPs are farmers [17],[18].

## 3. Food and health

Throughout the EU, cardiovascular diseases remain the leading cause of both disease and death, contributing 52% of all deaths and 23% of all disability adjusted life years [DALYs]; cancers come next, contributing a further 19% of all deaths [19],[20]. Unhealthy nutrition has been shown to include several risk factors for both cardiovascular diseases and cancers [21],[22]; indeed, the Interheart Study has demonstrated that nutrition-related risk factors are responsible for over 50% of all coronary heart disease deaths [23]. Moreover, dietary-related risk factors have been shown to be associated with 40% of DALYs globally [20].

Within the European Commission (EC), responsibility for both food safety and nutrition policies is located in the Public Health Directorate of Directorate General (DG) Sante (previously DG Sanco). However, there is obvious overlap with responsibilities of both DG Agriculture and Rural Development and DG Internal Market, Industry, Entrepreneurship, and Small Medium-sized Enterprises (SMSs), and policy development requires close liaison between these DGs, with some other DGs (e.g. for Research and Regional Policy) also being involved to a lesser extent [24].

## 4. Health challenges posed by the Common Agricultural Policy

It has already been demonstrated that the CAP promoted maximised production of beef and dairy products, both containing high levels of saturated fat; beef over-production has been one of the contributors to there being such a large number of burger bars on every high street (owing to the availability of large quantities of relatively cheap subsidised beef), while the dairy industry has worked hard to invent ever increasing numbers of attractive secondary dairy products, as a means of selling their high production. It is therefore unsurprising that Europe's populations have demonstrated high mean levels of serum LDL cholesterol [25].

Moreover, prior to 2008 the CAP traditionally never subsidised fruit and vegetable production [26], so there has rarely been over-production in this sector [16], except sometimes in summer months, leading to destruction of excess fruit (e.g. peaches) [27]. It has been calculated that, within the pre-2003 15 EU member states, annually approximately 5,000 CHD deaths and nearly 2,000 stroke deaths are attributable to inadequate fruit and vegetable production [28], and that if all CAP production subsidies were to be removed from saturated fat-rich farm production, this would save annually 7,000 CHD deaths and 2,000 stroke deaths [29]. Finland has demonstrated, largely before it became an EU member, the extent to which cardiovascular death rates can be cut radically, by (amongst other factors) cutting consumption of saturated fat-rich foods and by encouraging increased fruit and vegetable consumption [30], [31].

As already stated, sugar is another product the production of which is subsidised in the EU, although recent CAP reforms have included proposals for drastic reduction to sugar production subsidies (mainly so as no longer to distort the world sugar market prices). However, sugar consumption has been shown to be one of the prime causes of the increasing burden of child overweight and obesity. There is now discussion of the case for taxing sugar consumption [32]: this would be taxing the consumption of a food the production of which originally had been subsidised by the European tax payer.

## 5. Necessary reforms of agricultural policy

Since 2005 there have been a number of further reforms to the CAP, but these have been not been designed to promote any fundamental changes in its operation [33]. However, the European School Milk scheme, which was once designed to provide schools with high fat milk (while the EC also provided free saturated fat-rich butter to hospitals!), as a means of disposing of this excess dairy fat, which was the result of the population choosing increasingly to purchase low fat milk, was later modified in an attempt to address improving child nutrition, such that high fat milk consumption in schools was no longer encouraged [34]. More recently the European School Fruit Scheme has been added; in the 2014 reforms these two schemes have been combined, coordinated, provided with an increased budget, made subject to stricter nutritional criteria (referring to saturated fat and sugar content), and the incentives to encourage member states to adopt these schemes were increased [35].

In 2010, in a discussion document about future possible reforms, the EC emphasised the importance of public health nutrition, and stated that the CAP needed to contribute to this [36]. However, subsequent reforms have failed as yet to demonstrate any commitment to this stated priority. It remains the case that improving the public's health through healthier nutrition is still not in any way a stated objective of the CAP.

The need for reform of the CAP as an essential part of any comprehensive programme designed to reduce the incidence rates of chronic diseases, such as heart disease, diabetes and cancer, and to promote improved health and well-being of individuals as well as providing better welfare for society as a whole, has been demonstrated by Elinder et al. [36]. Accordingly, what is needed is reform along the following lines in the interests of future improved public health nutrition in Europe [7],[16],[37]:

- inserting improved public health nutrition for Europe's populations as a stated objective of the CAP;
- gradual phase-out of subsidy for all mammalian meat production (possibly an exception might be made for entirely grass-fed beef cattle, the meat from which is somewhat less saturated fat-rich);
- gradual phase-out of all subsidy for dairy production, other than for production of very low fat milk;
- gradual phase-out of all sugar production subsidies;
- inclusion of fish farming and production of vegetable protein products (e.g. pulses) within the whole farm subsidy payments scheme;
- more support to olive, rapeseed, etc., oil production;
- more social marketing of healthy foods throughout EU;
- introduction of production subsidies for fruit and vegetables;
- introduction of a healthy nutrition basic standard for farming (as for environmental protection, animal husbandry, and food safety).

It should be noted that many of these proposals coincide with the reforms to be required of agriculture in the interests of reduction in production of global warming gases (agriculture being the industry which leads production of these [38]): reduction in animal husbandry (especially of cows), and increased production of arable crops, would contribute significantly to reduction of release of global warming gases.

## 6. The Food Industry and Food Marketing

The nutritional risk factors for cardiovascular diseases, cancers, and obesity have been well known and understood now for some years, and yet these health problems remain prevalent. In the field of communicable diseases vectors are often found to be an essential component of epidemics, being the carriers of agents responsible for the aetiology of diseases, transporting them from human to human, or from animal to human. In the case of food, the food industry itself could be regarded as such a vector for disease [39], as a successful vector might be described as one which, while being a carrier of disease, also gives its victims some kind of limited satisfaction, at least in the short term

The food industry is in the business of making profits from selling foods. Having studied human taste, etc., and preferences, which were developed throughout evolution for times – unlike ours – when food was in short supply, and had to be found and hunted, the industry knows how to manufacture very attractive secondary food products (made from cheap agricultural raw materials), which contain high levels of sugar, salt, and saturated fat – in response to these preferences. These products provide the converse of healthy nutrition, yet they are advertised as contributing to increased human happiness, and sold as such. The advertising budgets of major international food firms are colossal: Nestle spent US$30.6 million on advertising in the USA in 2013 [40], while in 2012, world-wide Unilever spending on advertising totalled US$8.6 billion [41]. In 2012, 51 countries had GDPs of less than US$8.6 billion, equal to the Unilever advertising spend that year. [42].

In Europe governments should be in the business of regulating the food industry so that healthy nutrition (including obesity prevention) is protected and promoted. Unfortunately, these very powerful firms ensure by various means (e.g. support to political parties, etc.) that they maintain powerful influence over government food policies [43],[44]. Accordingly, the food corporations remain free to promote their most unhealthy products without constraint. Thus McDonalds and Coca-Cola were permitted to be the main sponsors of the London Olympic Games in 2012 [45]. Thus Coca-Cola is free to fund pseudo-science to investigate “what is driving the obesity epidemic”; Dr Steve Blair, who leads this research, says that “it's about being too darn lazy” [46]. Thus the UK Government confers, in the context of its Responsibility Deal, with representatives of Unilever, Coca-Cola, Mars, Mondelez, Nestle, and Premier Foods to discuss how best to promote healthy nutrition [47]. Similarly, in the context of the EU Platform on Diet, Physical Activity and Health, EC representatives discuss a similar agenda with other representatives of the international food industry [47]. Capewell et al. have described this denialism [48] as the SLEAZE process [49]:

- Scientific conspiracies are alleged (rather than admitting that each of these is based on a solid scientific consensus);
- Logical flaws in flaws in the arguments of the food industry (but which may initially sound plausible);
- Evidence severely selected to suit the case of the food industry, with all conflicting facts ignored;
- Absolute perfection demanded of public health advocates (e.g. “why has there been no RCT for passive smoking and cancer?”);
- Zany arguments and distractions, to draw attention away from the main issue (and which require the use of scarce public health resources to refute);
- Experts bought by the rich industries, to undermine good science, or to publish conveniently contradictory findings.

Meanwhile, TV programmes designed for children include (in their advertising slots) advertisements for foods likely to promote obesity; children, of all people, should be prevented from exposure to such material, so likely to be damaging to their longer term health as adults [50].

## 7. An agenda for public health nutrition advocacy

It is evident that thus far the EC (in particular DG Agriculture and Rural Development) has awarded much lower priority to healthy nutrition in Europe than it has to some of the other challenges it faces in relation to food production and distribution, and also that trade always seems to have priority over nutrition [51]. However, as public health can boast of so many successes in the interests of improved population health (e.g. safe drinking water, sanitation, slavery abolition, immunisation, road safety, seat belts, air pollution control, tobacco advertising bans, smoke-free legislation, etc.) [52], it is really quite surprising that has there been only such limited success so far in respect of attempts to improve healthy nutrition, including at EU level (the EU has powers to legislate in the field of nutrition since the implementation of the Amsterdam Treaty [53]; these powers were used to legislate for the creation of the European Food Safety Authority). Public health advocacy involving civil society has been very active at EU level [54],[55]. Smith in 2012 [56] and Hochschild in 2014 [57] have indicated the lessons in advocacy, learned from the slavery abolitionists, which it is suggested might be learned and adopted by public health advocates for improved population nutrition. An outline campaign strategy might be summarised thus:

- Describe clear vision of what needs to be achieved and of what can be done;
- Identify who should lead such a campaign, both at EU and member state levels;
- Develop a coalition of public health bodies, NGOs, etc., all working together under this common leadership to achieve commonly agreed goals;
- Identify principal enemies of this campaign, and identify means to repudiate the arguments they are likely to propagate;
- Develop an appropriate plan for phased interventions; these actions should be planned to be continuous and relevant on many fronts to the various necessary changes needed to achieve healthier nutrition;
- Identify from best science the means to support and justify all of these actions; however, this science must be presented in ways that the public can understand and appreciate, e.g. iconic pictures can be very effective in explaining complex points if used appropriately;
- Recruit political advocates (e.g. MEPs and MPs), who can be primed to ask appropriate political questions, based on evidence that can be provided to them.

It is evident that current nutrition in Europe, and the policies which influence this, are not conducive to good health [7], [16], and that thus far public health has not had much success in addressing these challenges (except in a few countries, such as Finland [31]). Public health has so many successes to be proud of; public health advocates must now resolve to achieve healthy nutrition in Europe, through reform of regulation of agriculture and of the food industry. Providing support to this aim must be a prime responsibility for every public health scientist and practitioner who is working to achieve healthier nutrition in Europe.
